# Radiobiological Meta-Analysis of the Response of Prostate Cancer to Different Fractionations: Evaluation of the Linear–Quadratic Response at Large Doses and the Effect of Risk and ADT

**DOI:** 10.3390/cancers15143659

**Published:** 2023-07-18

**Authors:** Juan Pardo-Montero, Isabel González-Crespo, Antonio Gómez-Caamaño, Araceli Gago-Arias

**Affiliations:** 1 Group of Medical Physics and Biomathematics, Instituto de Investigación Sanitaria de Santiago (IDIS), 15706 Santiago de Compostela, Spain; 2 Department of Medical Physics, Complexo Hospitalario Universitario de Santiago de Compostela, 15706 Santiago de Compostela, Spain; 3 Department of Applied Mathematics, Universidade de Santiago de Compostela, 15705 Santiago de Compostela, Spain; 4 Department of Radiation Oncology, Complexo Hospitalario Universitario de Santiago de Compostela, 15706 Santiago de Compostela, Spain; 5 Institute of Physics, Pontificia Universidad Católica de Chile, Santiago de Chile 7820436, Chile

**Keywords:** radiobiological modelling, radiotherapy, prostate cancer, LQ model, LQL model, meta-analysis, hypofractionation, SBRT

## Abstract

**Simple Summary:**

Prostate cancer is currently treated with different radiotherapy fractionations, including extreme hypofractionation. Some studies suggest that the response to large radiation doses per fraction may depart from the response predicted by the widely used linear–quadratic (LQ) model. In this study, we analysed a large dataset of dose–response data to evaluate departures from the LQ behaviour at large doses. In general, the response of prostate cancer to large doses of radiotherapy is best described by the LQ model, even though we observed some discrepancies at large doses for intermediate-risk patients, which merit further investigation. In addition, we characterised the radiobiological response of prostate cancer according to risk (low, intermediate, or high) and the addition or not of ADT to treatment.

**Abstract:**

The purpose of this work was to investigate the response of prostate cancer to different radiotherapy schedules, including hypofractionation, to evaluate potential departures from the linear–quadratic (LQ) response, to obtain the best-fitting parameters for low-(LR), intermediate-(IR), and high-risk (HR) prostate cancer and to investigate the effect of ADT on the radiobiological response. We constructed a dataset of the dose–response containing 87 entries/16,536 patients (35/5181 LR, 32/8146 IR, 20/3209 HR), with doses per fraction ranging from 1.8 to 10 Gy. These data were fit to tumour control probability models based on the LQ model, linear–quadratic–linear (LQL) model, and a modification of the LQ (LQ${}_{\mathrm{mod}}$) model accounting for increasing radiosensitivity at large doses. Fits were performed with the maximum likelihood expectation methodology, and the Akaike information criterion (AIC) was used to compare the models. The AIC showed that the LQ model was superior to the LQL and LQ${}_{\mathrm{mod}}$ models for all risks, except for IR, where the LQL model outperformed the other models. The analysis showed a low α/β for all risks: 2.0 Gy for LR (95% confidence interval: 1.7–2.3), 3.4 Gy for IR (3.0–4.0), and 2.8 Gy for HR (1.4–4.2). The best fits did not show proliferation for LR and showed moderate proliferation for IR/HR. The addition of ADT was consistent with a suppression of proliferation. In conclusion, the LQ model described the response of prostate cancer better than the alternative models. Only for IR, the LQL model outperformed the LQ model, pointing out a possible saturation of radiation damage with increasing dose. This study confirmed a low α/β for all risks.

## 1. Introduction

The response of prostate cancer to radiotherapy has been extensively analysed in the radiobiological modelling literature [1,2,3,4,5,6,7,8,9,10,11]. Most studies report a low α/β (typically in the 1–3 Gy range) and high sensitivity to fractionation, even though some studies suggest that the α/β may not be that low and the reported low values may be caused by hypoxia [8].

A low α/β for prostate cancer, lower than the α/β associated with the late toxicities of nearby tissues, may favour hypofractionated regimes. In recent years, stereotactic body radiotherapy (SBRT) has become widely used to treat many cancers [12], and several trials have explored the response/toxicity of hypofractionation in prostate cancer [13,14,15], with doses per fraction reaching up to 10 Gy.

The validity of the linear–quadratic (LQ) model for large dose fractions has been questioned [16,17,18]. Some studies point out a moderation of the LQ cell-killing effect with increasing dose, an effect that has been modelled with the linear–quadratic–linear (LQL) model and other approaches [19,20]. Furthermore, recent in vivo studies have shown an enhanced cell-killing effect at large doses attributed to indirect effects such as vascular damage and radiation-induced immune response [21,22,23], which has led to novel models including such effects [24,25,26,27].

Because the implementation of hypofractionation for prostate cancer is relatively new, there are not many radiobiological modelling studies investigating the response of prostate cancer to hypofractionation. We have to note two recent studies: Datta et al. [10] analysed eight isoeffective schedules (conventional and hypofractionated) and obtained a α/β value in the 1.3–8.2 Gy 95% confidence interval (CI); Vogelius and Bentzen [11] analysed 14 randomised trials of dose scalation and hypofractionation and obtained a tighter 95% CI of 1.3–2.0 Gy. Furthermore, a recent study by Royce et al. [28] analysed the tumour control probability (TCP) of 25 hypofractionated clinical studies and obtained the *EQD2* needed to reach 90–95% control by assuming α/β = 1.5 Gy.

In this work, we further explored the radiobiology of prostate cancer with a large dataset of treatments, with doses per fraction ranging from <2 Gy to 10 Gy. Our aim was two-fold: on the one hand, we evaluated whether the addition of dose–response data for severely hypofractionated schedules leads to deviations from the LQ model, by comparing the best fits obtained with the LQ model and other models. On the other hand, we determined the best-fitting radiobiological parameters that describe the response of prostate cancer to fractionation, split by risk level, in a large dataset containing a wide range of fractionations, and we investigated the effect of ADT on the radiobiological response.

## 2. Materials and Methods

### 2.1. Clinical Dataset

We analysed the dose–response data from 55 trials of prostate radiotherapy, building on data previously compiled in several radiobiological studies by Royce, Miralbell, Datta, Pedicini and colleagues [4,9,10,28] and reviewing recent articles from Pubmed. For each schedule, we extracted the number of patients, the distribution of patients with respect to the risk level, the number or percentage of patients receiving androgen deprivation therapy (ADT), the dose per fraction, the total dose, the treatment time, and the control at 5 years. Some studies included slightly different fractionations, and in those cases, the most-used fractionation was included. Control can be named differently in publications, but it generally refers to freedom from clinical or biochemical failure, with biochemical failure defined as PSA nadir + 2 ng/mL. We restricted our analysis to studies reporting Kaplan–Meier control values at 5 years: prostate cancer is usually a slow-growing disease, and differences in the control between different schedules may not be significant at 3 years. On the other hand, some studies also reported control at 7–7.5 years, but those data were discarded because there were very few of them. Kaplan–Meier control values were generally reported in the text, but sometimes were extracted from figures by using image analysis software (g3data, version 1.5.4). The original references are [4,14,29,30,31,32,33,34,35,36,37,38,39,40,41,42,43,44,45,46,47,48,49,50].

When separated by risk, we analysed 35, 32, and 20 schedules and 5181, 8146, and 3209 patients for low risk (LR), intermediate risk (IR), and high risk (HR), respectively. Some studies included extra groups, such as “favorable intermediate risk”, “unfavorable intermediate risk”, and “very low risk”. In such cases, those results were merged into a single group weighting with the number of patients (favourable and unfavourable intermediate risk merged in “intermediate risk”; very low risk and low risk merged in “low risk”).

Several of the clinical protocols included androgen deprivation therapy (ADT). In general, LR patients did not receive ADT; some schedules for IR patients included ADT; a majority of HR patients received ADT. For IR and HR patients, we also analysed separately schedules that included ADT for most patients (≥50%) and those that did not: 9/32 IR and 15/20 HR schedules included ADT according to this definition.

An overview of the schedules included in the analysis is presented in Table 1, and further detailed information is presented in Table S1.

### 2.2. Radiobiological Modelling: Dose–Response

We relied on the LQ model to fit the dose–response. The surviving fraction of tumour cells after a dose *d* is

(1) $$\log{\mathrm{SF}}_{\mathrm{LQ}}=-\alpha d-\beta d^{2}$$

with α and β being the linear and quadratic parameters of the LQ model.

The LQL model [19], which includes a moderation of the LQ-predicted cell death with increasing dose, characterised by the parameter δ, was also investigated:

(2) $$\log{\mathrm{SF}}_{\mathrm{LQL}}=-\alpha d-\frac{2\beta }{\delta^{2}}\left(\delta d+\exp\left(-\delta d\right)-1\right)$$

In addition, we investigated an ad hoc modification of the LQ model presented in [26], which includes an increasing effective β term with increasing dose to account for indirect cell damage at large doses, an effect that is characterised by a parameter *b*:

(3) $$\log{\mathrm{SF}}_{\mathrm{LQ}}=-\alpha d-\beta \left(1+b\sqrt{d}\right)d^{2}$$

When delivering a treatment of *n* fractions, the overall surviving fraction is given by:

(4) $${\mathrm{SF}}_{\mathrm{treat}}=\left(\prod_{i=1}^{n}{\mathrm{SF}}_{i}\right)\exp\left(\lambda \max\left(0,T-T_{k}\right)\right)$$

where ${\mathrm{SF}}_{i}$ is the surviving fraction associated with each fraction, *T* is the treatment time, and proliferation is modelled as exponential with rate λ after a kick-off time $T_{k}$.

The tumour control probability was modelled using a logistic function [51]:

(5) $$\mathrm{TCP}=\frac{1}{1+{\left(\frac{D_{50}}{\mathrm{EQD}𝟤}\right)}^{4\gamma_{50}}}$$

where $D_{50}$ is the dose corresponding to 50% control (in 2 Gy fractions) and $\gamma_{50}$ is the normalised dose–response gradient. *EQD2* is the equivalent dose in 2 Gy fractions of a given schedule, which is model-dependent. For example, for the LQ model, it can be calculated as:

(6) $$\mathrm{EQD}{𝟤}_{\mathrm{LQ}}=\frac{D+\frac{dD}{\alpha /\beta }-\frac{\lambda }{\alpha }\max\left(0,T-T_{k}\right)}{\left(1+\frac{2}{\alpha /\beta }\right)}$$

where *D*, *d*, and *T* are the total dose, dose per fraction, and treatment time of the radiotherapy schedule. Similar equations can be written for the LQL and LQ${}_{\mathrm{mod}}$ models using Equations (2) and (3):

(7) $$\mathrm{EQD}{𝟤}_{\mathrm{LQL}}=\frac{D+\frac{2(\delta d+\exp\left(-\delta d\right)-1)D}{\left(\alpha /\beta \right)d\delta^{2}}-\frac{\lambda }{\alpha }\max\left(0,T-T_{k}\right)}{\left(1+\frac{2\delta +\exp\left(-2\delta \right)-1}{\left(\alpha /\beta \right)\delta^{2}}\right)}$$

(8) $$\mathrm{EQD}{𝟤}_{{\mathrm{LQ}}_{\mathrm{mod}}}=\frac{D+\frac{dD(1+b\sqrt{d})}{\alpha /\beta }-\frac{\lambda }{\alpha }\max\left(0,T-T_{k}\right)}{\left(1+\frac{2(1+\sqrt{2}b)}{\alpha /\beta }\right)}$$

### 2.3. Statistical Methods

Fitting was performed by using the maximum likelihood methodology, assuming binomial statistics for the reported control values. The optimisation (minimisation of the −logL function, where *L* is the likelihood) was performed with an in-house-developed simulated annealing algorithm.

The free parameters of the fit are α/β, λ/α, $T_{k}$, $\gamma_{50}$, and $D_{50}$ for the LQ model. For the LQL and LQ${}_{\mathrm{mod}}$ models, there is an extra parameter, δ and *b*, respectively. Notice that, in this fit, the value of α cannot be determined, only α/β (which conditions the response to different fractionation). The proliferation rate cannot be determined either, as it is entangled with α. We define $\lambda^{\prime }=\lambda /\alpha$, which has units of Gy/day, and it is related to the dose needed to compensate for repopulation.

The profile likelihood method was used to obtain 95% confidence intervals (CIs) of the best-fitting parameters [52,53]. The implementation of the profile likelihood method is presented in more detail in the Supplementary Materials, including Figure S1.

The Akaike information criterion with sample size correction was used to rank different models [54]. The $AIC_{c}$ is given by:

(9) $${\mathrm{AIC}}_{c}=-2\log L+2k+\frac{2k(k+1)}{S-k-1}$$

where *k* is the number of parameters of the model, *S* is the sample size, and *L* is the maximum of the likelihood function. Models with lower $AIC_{c}$ are preferred. In this regard, $\Delta AIC_{c}$ is defined as

(10) $$\Delta AIC_{c}^{\mathrm{model}}=AIC_{c}^{\mathrm{ref}}-AIC_{c}^{\mathrm{model}}$$

where $AIC_{c}^{\mathrm{ref}/\mathrm{model}}$ refers to the ${\mathrm{AIC}}_{c}$ of the reference model (the LQ model in this work) and the model under study.

The implementation of the methodology was performed in Matlab (Mathworks, Natick, MA, USA).

### 2.4. Radiobiological Modelling: α and Number of Clonogens

Some further information on the radiobiology of the tumours can be obtained from the analysis of the best-fitting parameters. Combining the TCP Poisson formulation [55] and the definition of $D_{50}$, we can write (using the LQ model)

(11) $$\mathrm{TCP}\left(D_{50}\right)=0.5=\exp\left(-N\times SF\left(D_{50}\right)\right)=\exp\left(-N\exp\left(-\alpha \left(D_{50}+\frac{2D_{50}}{\alpha /\beta }\right)\right)\right)$$

We can use here the definition of biologically equivalent dose (BED) [56] to calculate the BED associated with $D_{50}$ as:

(12) $${\mathrm{BED}}_{50}=D_{50}+\frac{2D_{50}}{\alpha /\beta }$$

Developing Equation (11), we obtain:

(13) $$N=\exp\left(-0.37+\alpha {\mathrm{BED}}_{50}\right)≃\exp\left(\alpha {\mathrm{BED}}_{50}\right)⟹\frac{\log N}{\alpha }≃BED_{50}$$

This expression provides a qualitative relationship between the number of clonogen cells (*N*), their radiosensitivity (α), and $D_{50}$ (obtained from the fit to the dose–response data). Notice that, for simplicity, we ignored the radiosensitivity averaging methodology, which is usually included in the computation of TCP values with the Poisson model; thus, Equation (13) has to be taken as a simple qualitative approximation.

## 3. Results

In Table 2, we present the best-fitting parameters and the goodness-of-fit (−logL and $AIC_{c}$) obtained with the LQ, LQL, and LQ${}_{\mathrm{mod}}$ models for low, intermediate, and high risk. For IR (HR), we also present separately the fits for schedules that did not include ADT (included ADT).

For LR and HR, the best fits obtained with the LQL model have δ∼0, and therefore, the best-fitting solutions are almost identical to those obtained with the LQ model. Because the LQL model has one extra degree of freedom, this results in higher $AIC_{c}$ than those obtained with the LQ ($\Delta AIC_{c}$ < 0) model. For IR, the LQL model clearly outperformed the LQ (and LQ${}_{\mathrm{mod}}$) model, with $\Delta AIC_{c}≃$ 36 (Table 2 and Figure S2). On the other hand, the best fits obtained with the LQ${}_{\mathrm{mod}}$ model showed a very modest improvement over the LQ model for LR and HR when comparing the likelihood, but due to the extra parameter, this did not lead to $\Delta AIC_{c}$ > 0.

The best-fitting parameters obtained with the LQ model are presented in more detail in Table 3, including the 95% CIs. The results for intermediate and high risk are also presented separately for cohorts including/not including ADT as part of the treatment. The best fits showed low α/β values (2.0 Gy for LR, 3.4 Gy for IR, and 2.8 Gy for HR), while the 95% CIs were [1.7–2.3] Gy for LR, [3.0–4.0] Gy for IR, and [1.4–4.2] Gy for HR. The $D_{50}$ values ranged from 56.2 Gy for LR to 59.8 Gy for HR. The results showed no proliferation for LR tumours and proliferation rate (kick-off time) values of 0.41 Gy/day (24 days) for IR and 0.35 Gy/day (21 days) for HR. It is important to notice that we implemented a minimum constraint of 21 days for $T_{k}$.

When analysing separately the data for IR/HR patients that were treated with ADT or not, we obtained α/β = 2.8 Gy, $D_{50}$ = 58.1 Gy, $\lambda^{\prime }$ = 0.32 Gy/day, $T_{k}$ = 21 days for IR “only RT”, and α/β = 2.1 Gy, $D_{50}$ = 58.5 Gy and no proliferation for HR “RT+ADT”. The best fits for IR “RT+ADT” and HR “only RT” are also presented in Table 3, but due to the low number of schedules involved (9 and 5, respectively), the confidence intervals are very wide.

**Figure 1:**
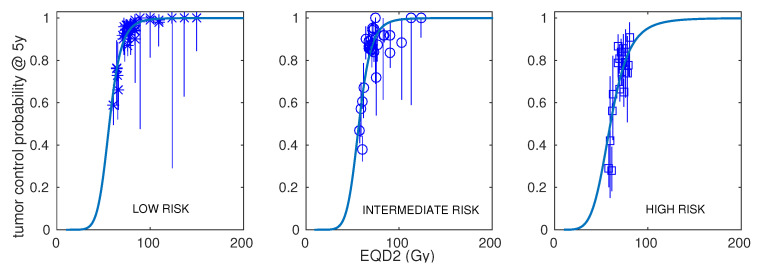
Best fits to prostate carcinoma dose–response data obtained with the linear–quadratic model. Results are presented separately for low risk (**left** panel), intermediate risk (**central** panel), and high risk (**right** panel).

**Figure 2:**
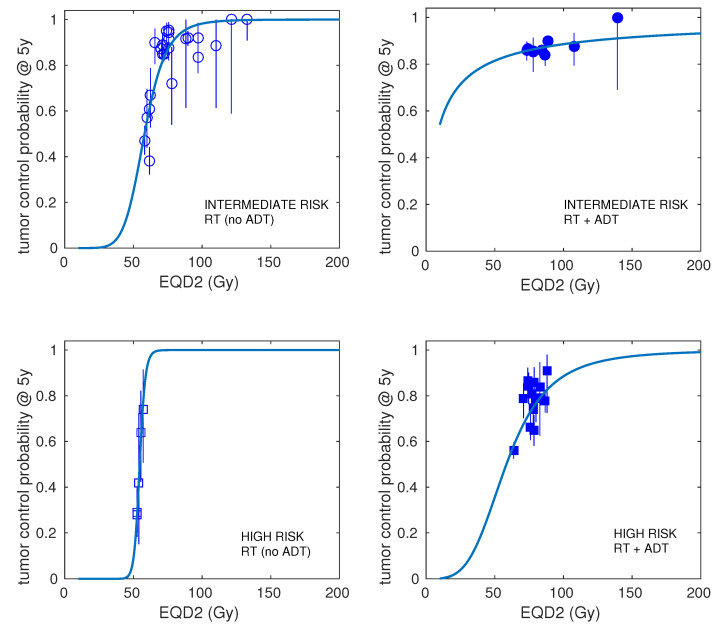
Best fits to intermediate- and high-risk prostate carcinoma dose–response data obtained with the linear–quadratic model. Results are presented separately for cohorts that used androgen deprivation therapy (ADT) and cohorts that did not use ADT in addition to radiotherapy.

In Figure 1, we show the best fits to the prostate carcinoma dose–response data obtained with the LQ model. The results are presented separately for LR, IR, and HR. In Figure 2, the best fits for IR and HR are shown separately for cohorts including ADT and cohorts not using ADT in addition to radiotherapy.

**Figure 3:**
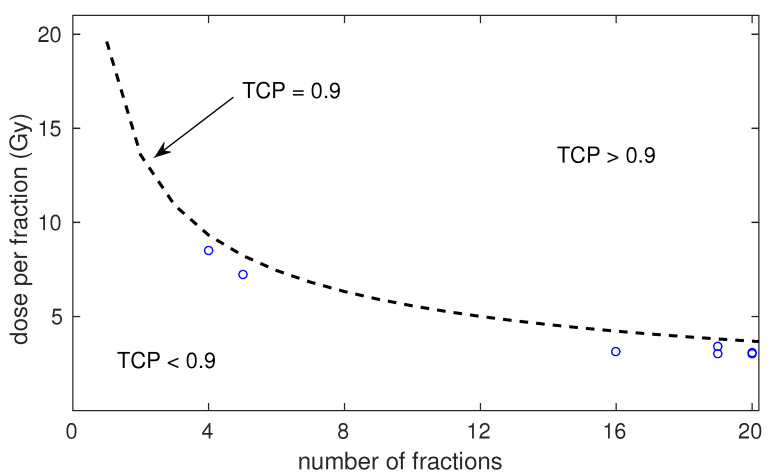
Modelled dose per fraction versus number of fractions to achieve 90% control for HR patients treated with radiotherapy and ADT (dashed line). The circles represent the experimental fractionations included in the dataset.

We investigated the dose per fraction versus number of fractions that would be necessary to obtain 90% control for HR patients treated with radiotherapy and ADT according to the best-fitting parameters obtained with the LQ model. These results are presented in Figure 3, where we also present the experimental fractionations included in the dataset for “RT+ADT”.

Applying Equations (11) and (13), which qualitatively link the number of clonogens and the radiosensitivity of the tumour cells, to the best-fitting parameters obtained with the LQ model, we obtain:

(14) $$\frac{\log N}{\alpha }=\left\{\begin{array}{cc} 112.2\;\mathrm{Gy} & \left(\mathrm{LR}\right)\\ 89.9\;\mathrm{Gy} & \left(\mathrm{IR}\right)\\ 101.4\;\mathrm{Gy} & \left(\mathrm{HR}\right)\\ 99.2\;\mathrm{Gy} & \left(\mathrm{IR}\;\mathrm{no}\;\mathrm{ADT}\right)\\ 114.4\;\mathrm{Gy} & \left(\mathrm{HR}\;\mathrm{no}\;\mathrm{ADT}\right) \end{array}\right.$$

If we assume $N_{LR}$ < $N_{IR}$ < $N_{HR}$ (which is supported by the analysis of Pedicini et al. [9], who reported $N_{LR}$ = 4.5 × 10${}^{5}$, $N_{IR}$ = 3 × 10${}^{6}$, $N_{HR}$ = 2 × 10${}^{7}$), we may conclude that LR cells might be less radiosensitive than HR/IR cells (by using the numbers of cells reported in [9], we obtained $\alpha_{LR}∼0.12$ Gy${}^{-1}$, versus $\alpha_{IR/HR}∼$ 0.17 Gy${}^{-1}$).

When including schedules from different studies, they will most likely use different definitions of the PTV (different margins), different cost functions (resulting in different dose homogeneity in the PTV), and different treatment modalities (CRT, IMRT), which can increase the uncertainties of the analysis.

## 4. Discussion

In this study, we investigated the dose–response of prostate cancer from a dataset containing 87 entries/16,536 patients (35/5181 low risk, 32/8146 intermediate risk, 20/3209 high risk), with doses per fraction ranging from 1.8 to 10 Gy. Rather than analysing independently trials reporting control for different fractionations (the approach followed in [10,11]), we analysed a dataset containing studies from different trials, like in [4,26,57]. Our approach increased the heterogeneity of the dataset and, therefore, may increase the uncertainties of the analysis (different studies may use different margins, different dose homogeneity constraints on the PTV, different dose calculation algorithms, etc.), which constitutes a limitation of the present study. On the other hand, this allowed us to investigate the dose–time response (obtaining proliferation parameters) and to evaluate models with more degrees of freedom, which may not be possible with the former approach. The large dataset also allowed analysing separately different risk levels and the use of ADT.

It has been suggested that the LQ model may fail to describe the dose–response at large doses per fraction due to the contribution of effects such as damage repair, vascular damage, or radiation-induced immune effects [16,18]. Therefore, we investigated not only the LQ model, but also other models that include departures from the LQ behaviour at large doses per fraction (the LQL model, with decreasing radiosensitivity with increasing dose, and a phenomenological modification of the LQ model, with increasing radiosensitivity with increasing dose). Fits with the LQ${}_{\mathrm{mod}}$ model showed a very modest improvement over the LQ model for LR and HR ($\Delta AIC_{c}∼$0.1). Analyses based on the AIC typically set stronger thresholds, demanding $\Delta AIC_{c}$ > 6 to state the superiority of a given model over another [58]. On the other hand, fits with the LQL model showed a clear improvement over the LQ model for IR patients ($\Delta AIC_{c}$ > 30).

The superiority of the LQL model over the LQ model for IR merits further discussion. Interestingly, the study of Vogelius and Bentzen [11] found a similar pattern with increasing dose per fraction when analysing a dataset of studies not separated by risk. An analysis of the schedules included in the dataset showed that the superiority of the LQL model in our analysis was strongly conditioned by a schedule reported in a recent study by Levin-Epstein et al. [44]. In that work, they reported control for 1904 patients treated with SBRT, including 157 intermediate-risk patients (93 favourable, 64 unfavourable) treated with 38 Gy in four fractions (9.5 Gy per fraction). Control at 5 years for those patients was 83.6% (86.7% for favourable and 79.2% for unfavourable), well below the control obtained in the same risk group for 35 Gy/5f (89.0%), 36.25 Gy/5f (95.2%), and 40 Gy/5f (92.0%). If we exclude the 38 Gy/4f results from the analysis, the $\Delta AIC_{c}$ for the LQL decreased from 36 to 6. In our dataset, there were schedules delivering similar doses per fraction that reported higher control, but they included a much lower number of patients (e.g., 38 Gy/4f, control = 92%, 39 patients). The relatively low control rates obtained for a dose per fraction of 9.5 Gy may be a hint of the LQL behaviour at large doses, but should be confirmed by more experimental studies.

In addition, the fact that the superiority of the LQL model was observed only for IR may be related to the poor goodness-of-fit obtained for IR (−logL > 200 vs. −logL∼ 100 for LR/HR). The worse fits obtained for IR could be caused by a more-heterogeneous dataset (caused by different ratios of favourable/unfavourable IR patients or more heterogeneity in the administration of ADT).

Another limitation of the present study was that we only analysed a limited number of dose–response models. We cannot discard that other models may provide a better fit to the experimental data. For example, models accounting for hypoxia and reoxygenation, which have been suggested to play a role in the response of prostate cancer [8,59], have not been investigated. In this regard, the large dataset that we assembled (Table S1) may prove useful for other researchers to investigate different models.

The analysis based on the LQ model supports a low α/β value for all risk groups of prostate cancer, with 95% CI of [1.7–2.3] Gy for LR, [3.0–4.0] Gy for IR, and [1.4–4.2] Gy for HR. Nonetheless, our analysis showed that the α/β of IR was larger than that of LR, which may be taken into account when designing optimal fractionations. The low α/β values were in general agreement with several radiobiological analyses of the dose–response in prostate cancer [1,2,3,4,5,6,7,9,10,11]. However, most of these studies did not include hypofractionated treatments (only [10,11,28]) and/or analysed a lower number of schedules.

High-risk, and to a lesser extent intermediate-risk, prostate cancer is usually treated with a combination of radiotherapy and ADT. When analysing separately HR cohorts including ADT or not, it seemed that the addition of ADT eliminated tumour proliferation ($\lambda^{\prime }$ = 0 Gy/day for HR cohorts including ADT versus $\lambda^{\prime }$ = 0.35 Gy for all HR cohorts). It would be of interest to know whether the addition of ADT affects the α/β of the tumour. However, due to the low number of HR schedules that did not include ADT (and IR schedules that included ADT), the confidence intervals were very wide, and no conclusive evidence can be reported on the differences between adding ADT or not.

Control rates for LR and IR prostate cancer are typically above 90%. However, control rates for HR prostate cancer are lower. We investigated the dose per fraction that was necessary to obtain 90% for HR patients treated with radiotherapy and ADT according to the best-fitting parameters obtained with the LQ model. The experimental schedules included in the dataset were below the TCP = 0.9 boundary (see Figure 3). According to the model, doses per fraction of 10.9 Gy, 8.2 Gy, and 5.6 Gy are needed to reach 90% control with 3, 5, and 10 fractions. Whether the toxicity associated with such a dose escalation is tolerable was not studied in this work. It may be worth exploring hypofractionated dose escalation schedules aiming at increasing the control rate of HR cancer for subsets of patients who are genetically less-predisposed to suffer toxicity [60].

## 5. Conclusions

In conclusion, the analysis of the dose–response of prostate cancer did not show evidence of effects beyond the LQ model contributing at large doses per fraction, except for IR schedules where the LQL is superior to the LQ, pointing out a possible moderation of radiosensitivity with increasing dose. This behaviour has been observed in a previous study [11] and merits further investigation because it might affect the dose prescription in prostate SBRT. Our analysis showed a low α/β for all risks of prostate cancer. However, the α/β for IR (95% CI [3.0–4.0] Gy) was significantly larger than for LR (95% CI [1.7–2.3] Gy). In addition, the best fits did not show proliferation for LR and moderate proliferation for IR/HR, and proliferation was suppressed when adding ADT to the treatment.

## Figures and Tables

**Table 1:** Overview of the characteristics of the schedules included in the analysis.

Risk	Number of Schedules	Number of Patients (Range)	Dose per Fraction (Range)	Total Dose (Range)	Treatment Time (Range)	ADT (Fraction of Schedules)	Control at 5 Years (Range)
LR	35	3–550	1.8–10 Gy	33.5–81 Gy	3–62 days	3/35	0.59–1.00
IR	32	7–839	1.8–10 Gy	34–81 Gy	3–62 days	9/32	0.38–1.00
HR	20	12–812	1.8–8.5 Gy	34–81 Gy	3–62 days	15/20	0.28–0.908

**Table 2:** Best fits obtained with the LQ, LQL, and LQmod models to prostate carcinoma dose–response data, separated by risk (low, intermediate, and high risk). For intermediate risk, results are also presented separately for schedules not including ADT. For high risk, results are also presented separately for schedules including ADT. The table shows the best-fitting parameters, maximum likelihood, and $AIC_{c}$ values. Improvements on the performance of the LQ model ($\Delta AIC_{c}$ > 0) are highlighted in bold and italics. The symbol * indicates that the best-fitting parameter reached the edge of the constraint window.

Risk	Model	Parameters									
		α/β	$\lambda^{\prime }$	$T_{k}$	δ	b	$D_{50}$	$\gamma_{50}$	−logL	${\mathrm{AIC}}_{c}$	$\Delta {\mathrm{AIC}}_{c}$
		**(Gy)**	**(Gy day** ${}^{-1}$ **)**	**(day** ${}^{-1}$ **)**	**(Gy** ${}^{-1}$ **)**	**(Gy** ${}^{-1/2}$ **)**	**(Gy)**				
LR	LQ	2.0	0.00	-	-	-	56.2	2.17	89.4	190.8	-
	LQL	2.0	0.00	-	0.00	-	56.2	2.17	89.4	193.7	−2.9
	LQ${}_{\mathrm{mod}}$	2.6	0.00	-	-	0.07	55.8	2.11	89.2	193.4	−2.6
IR	LQ	3.4	0.41	24.0	-	-	56.9	2.14	220.9	454.2	-
	LQL	0.4	0.00	-	0.28	-	62.8	2.18	201.3	418.0	***36.2***
	LQ${}_{\mathrm{mod}}$	3.5	0.41	23.9	-	0.00	56.8	2.15	220.9	457.2	−3.0
HR	LQ	2.8	0.35	21.0 *	-	-	59.8	1.45	105.0	224.4	-
	LQL	2.8	0.35	21.0 *	0.00	-	59.8	1.45	105.0	228.5	−4.2
	LQ${}_{\mathrm{mod}}$	11.2	0.34	21.0 *	-	0.75	58.7	1.47	103.9	226.2	−1.8
IR	LQ	2.8	0.32	21.0 *	-	-	58.1	1.85	157.6	328.7	-
(no ADT)	LQL	0.5	0.00	-	0.24	-	63.6	2.01	138.7	294.6	***34.1***
	LQ${}_{\mathrm{mod}}$	2.8	0.32	21.0 *	-	0.00	58.1	1.85	157.6	332.4	−3.7
HR	LQ	2.1	0.00	-	-	-	58.5	0.95	72.6	161.8	-
(ADT)	LQL	2.1	0.00	-	0.00	-	58.5	0.95	72.5	167.6	-5.8
	LQ${}_{\mathrm{mod}}$	18.7	0.00	-	-	1.99	56.8	0.88	71.2	164.9	−3.1

**Table 3:** Best-fitting parameters and 95% confidence intervals (within parentheses) of prostate carcinoma dose–response data obtained with the linear–quadratic model. Results are separated by risk and for intermediate and high risk are also presented separately for schedules that included or did not include ADT. Data for IR with ADT and HR with no ADT are shown only for illustrative purposes, because, due to the low number of schedules, the confidence intervals are very wide.

	α/β (Gy)	$\lambda^{\prime }$ (Gy day${}^{-1}$)	$T_{k}$ (day${}^{-1}$)	$D_{50}$ (Gy)	$\gamma_{50}$
LR	2.0	0	-	56.2	2.17
	(1.7, 2.3)	(0, 0.13)		(54.4, 58.0)	(1.90, 2.47)
IR	3.4	0.41	24.0	56.9	2.14
	(3.0, 4.0)	(0.31, 0.49)	(21.0, 25.5)	(55.5, 57.9)	(1.92, 2.40)
HR	2.8	0.35	21.0	59.8	1.45
	(1.4, 4.2)	(0, *∞*)	(21.0, *∞*)	(57.1, 63.9)	(1.07, 1.83)
IR	2.8	0.32	21.0	58.1	1.85
(no ADT)	(2.1, 3.5)	(0.09, 0.46)	(21.0, 27.3)	(56.5, 60.0)	(1.55, 2.14)
HR	2.1	0	-	58.5	0.95
(ADT)	(1.5, 3.5)	(0, 0.31)		(54.3, 61.5)	(0.75, 1.25)
IR	0.1	0	-	8.1	0.20
(ADT)	(0, *∞*)	(0, *∞*)		(0.4, 40.5)	(0.11, 0.80)
HR	100.0	3.31	39.9	54.6	6.68
(no ADT)	(7.1, *∞*)	(1.09, *∞*)	(21.0, 40.8)	(49.8, 60.5)	(2.70, 10.67)

## Data Availability

All data generated or analysed during this study are included in this published article and in the Supplementary Materials.

## Supplementary Materials

The following Supporting Information can be downloaded at: https://www.mdpi.com/article/10.3390/cancers15143659/s1, Figure S1: Illustration of the calculation of 95% confidence intervals for the parameter α/β in IR patients; Figure S2: Best fits to intermediate risk dose–response data for prostate cancer obtained with the linear–quadratic (LQ) and linear–quadratic–linear (LQL) models; Table S1: Detailed information of the analysed schedules for low- (LR), intermediate- (IR), and high-risk (HR) prostate cancer, including: number of patients (N), dose per fraction (d), number of fractions (n), total dose (D), overall treatment time (OTT), percentage of patients receiving ADT, control at five years, and the first author and year of the study.
