# Supplementary material for: Radiobiological Meta-Analysis of the Response of Prostate Cancer to Different Fractionations: Evaluation of the Linear–Quadratic Response at Large Doses and the Effect of Risk and ADT
Source: Cancers (Basel). 2023 Jul 18;15(14):3659. doi: 10.3390/cancers15143659 (PMC10377316; doi:10.3390/cancers15143659)
Supplement: Supplementary file 1 [file cancers-15-03659-s001.zip › cancers-2437559-supplementary.pdf]

**Supplementary Materials for *"Radiobiological meta-analysis of the response of prostate cancer to different fractionations: evaluation of the linear-quadratic response at large doses, and the effect of risk and ADT"***

Juan Pardo-Montero, Isabel González-Crespo, Antonio Gómez-Caamaño, Araceli Gago-Arias

## 1. Statistical methods

The profile likelihood method was used to obtain confidence intervals of best-fitting parameters. A parameter value $\theta_0$ will be in the (1-α) confidence interval if it verifies

$$2\left[\log L_1(\theta_0) - \log L_{opt}\right] < \chi^2_{1-\alpha}(1)/2 \ , \qquad \text{(S1)}$$

where log $L_{opt}$ is the likelihood of the full model, log $L_1(\theta_0)$ is the likelihood of the model when the value of the parameter $\theta$ is set to $\theta_0$, and $\chi^2_{1-\alpha}(1)$ is the $(1-\alpha)$ quantile of a $\chi^2$ distribution with 1 d.f.

In order to obtain confidence intervals for a given parameter $\theta$, we evaluated equation (SM1) for a set of $\{\theta_0\}$ values, typically 8-12, paying attention to having values off the 95% confidence interval on both the left and right side. The set of $\{\theta_0$ , log $L_1(\theta_0)$-log $L_{opt}\}$ were interpolated with a shape-preserving piecewise cubic hermite interpolating polynomial (pchip), and the specific values defining the left ($\theta_L$) and right ($\theta_R$) sides of the confidence interval, verifying

$$2\left[\log L_1(\theta_{L/R}) - \log L_{opt}\right] = \chi^2_{1-\alpha}(1)/2 \qquad \text{(S2)}$$

were obtained from the interpolation.

The profile likelihood method was implemented with one particularity: If best-fitting parameters showed no proliferation, the confidence interval of the proliferation rate $\lambda'$ was obtained by fixing $T_k$ = 21 days (the minimum kick-off time allowed in this study). Fixing the value of $T_k$ is necessary, otherwise the optimizer will obtain optimal non-proliferative solutions, equivalent to $\lambda'$=0, by selecting large values of $T_k$ (and therefore 2[log $L_1(\lambda')$ - log $L_{opt}$]=0 for all $\lambda'$).

In Supplementary Figure 1 we illustrate this calculation for the $\alpha/\beta$ parameter in IR.

## 2. Supplementary Figures

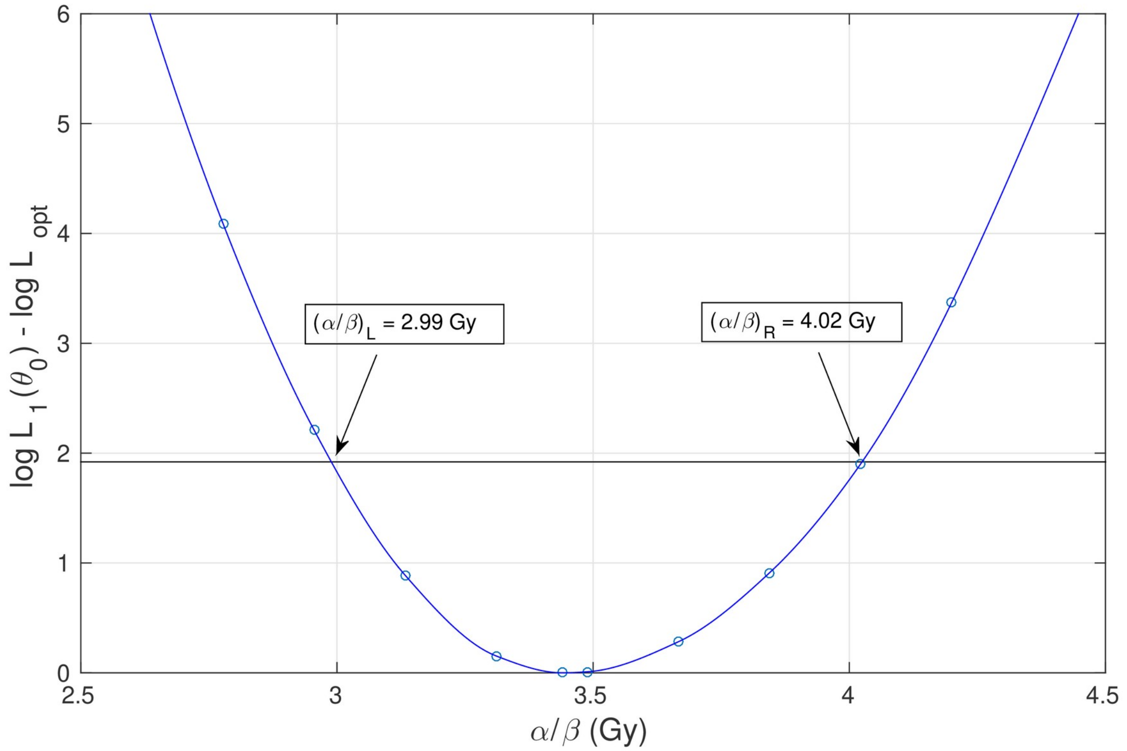


**Figure S1**: Illustration of the calculation of 95% confidence intervals for the parameter (α/β) in IR patients.

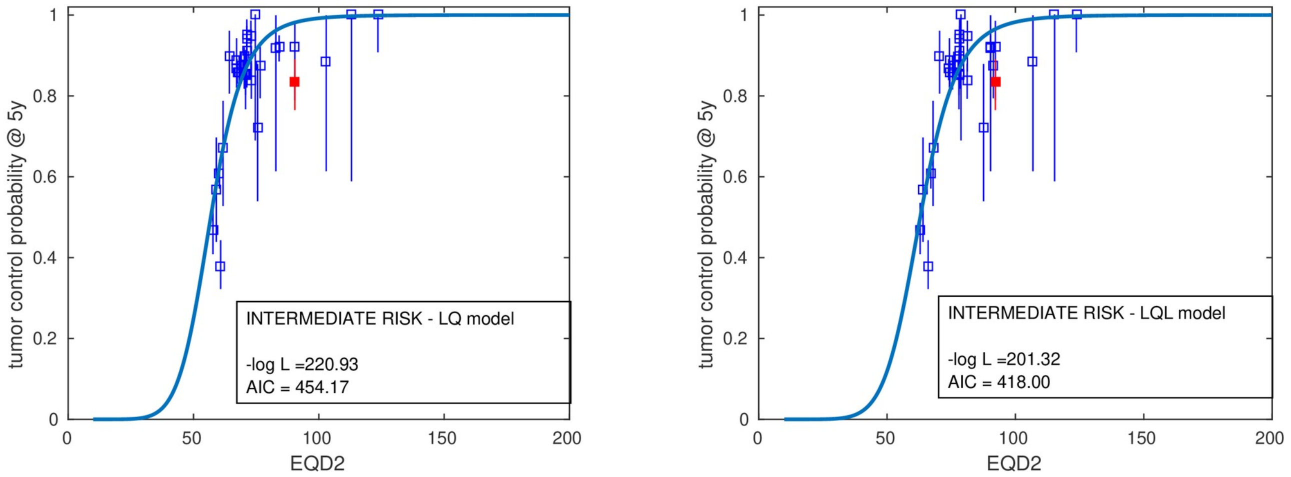


**Figure S2:** Best fits to intermediate risk dose-response data for prostate cancer obtained with the linear quadratic (LQ) and linear-quadratic-linear (LQL) models: left panel and right panel, respectively. In red we highlight the schedule that conditions the superiority of the LQL model: reported by Levin-Epstein *et al.*, it includes 157 intermediate risk patients treated with 38 Gy in 4 fractions (9.5 Gy per fraction), and control at 5 years was 83.6%.

## 3. Supplementary Tables

**Table S1:** Detailed information of the analyzed schedules for low (LR), intermediate (IR), and high risk (HR) prostate cancer, including: number of patients (N), dose per fraction (d), number of fractions (n), total dose (D), overall treatment time (OTT), percentage of patients receiving ADT, control at five years, and the first author and year of the study.

| Risk | N | d (Gy) | n | D (Gy) | OTT (days) | ADT (%) | Control 5 years | Reference |
|---|---|---|---|---|---|---|---|---|
| LR | 189 | 7.25 | 5 | 36.25 | 7 | 5.3 | 0.990 [a] | Davis2015 [33] |
| LR | 45 | 7.25 | 5 | 36.25 | 7 | 0 | 0.977 [b] | McBride2012 [48] |
| LR | 3 | 9 | 5 | 45 | 0 | 0 | 1 [c] | Hannan2016 [36] |
| LR | 8 | 9.5 | 5 | 47.5 | 0 | 0 | 1 [c] | Hannan2016 [36] |
| LR | 22 | 10 | 5 | 50 | 0 | 0 | 1 [c] | Hannan2016 [36] |
| LR | 40 | 6.7 | 5 | 33.5 | 4 | 0 | 0.930 [d] | Madsen2007/Miralbell2012 [46, 4] |
| LR | 84 | 7 | 5 | 35 | 29 | 1.2 | 0.975 [c] | Alayed2018 [29] |
| LR | 102 | 8 | 5 | 40 | 10 | 0 | 1 [c] | Mantz2014 [47] |
| LR | 40 | 9.5 | 4 | 38 | 0 | 0 | 0.980 [e] | Fuller2014 [35] |
| LR | 67 | 7.25 | 5 | 36.25 | 7.7 | 0 | 0.940 [aa] | King2012 [40] |
| LR | 5 | 8.5 | 4 | 34 | 3 | 0 | 1 [c] | Kang2011 [38] |
| LR | 324 | 7.25 | 5 | 36.25 | 4 | 0* | 0.965 [a] | Katz2016 [39] |
| LR | 61 | 7.25 | 5 | 36.25 | 9.7 | 11.5 | 0.944 [bb] | Bernetich2014 [31] |
| LR | 422 | 3.13 | 16 | 50 | 21 | 26.3 | 0.764 [e] | Miralbell2012 (Logue data) [4] |
| LR | 21 | 4 | 14 | 56 | 28 | 0 | 0.900 [e] | Miralbell2012 [4] |
| LR | 75 | 2 | 39 | 78 | 52 | 7.1 | 0.953 [e] | Kupelian2007/Miralbell2012 [42, 4] |
| LR | 257 | 2.5 | 28 | 70 | 37 | 29.8 | 0.950 [e] | Kupelian2007/Miralbell2012 [42, 4] |
| LR | 113 | 2 | 33 | 66 | 44 | 0 | 0.660 [e] | Lukka2005/Miralbell2012 [45, 4] |
| LR | 113 | 2.63 | 20 | 52.5 | 27 | 0 | 0.590 [e] | Lukka2005/Miralbell2012 [45, 4] |
| LR | 34 | 2 | 32 | 64 | 43 | 0 | 0.760 [e] | Yeoh2006/Miralbell2012 [49, 4] |
| LR | 26 | 2.75 | 20 | 55 | 27 | 0 | 0.730 [e] | Yeoh2006/Miralbell2012 [49, 4] |
| LR | 275 | 1.8 | 45 | 81 | 62 | 0** | 0.922 [aa] (51 months) | Zelefsky2002 [50] |
| LR | 247 | 2 | 38 | 76 | 51 | 21.1 | 0.869 [e] | Miralbell2012 (Leborgne data) [4] |
| LR | 30 | 1.8 | 42 | 75.6 | 57 | 0*** | 0.960 [c] | Kuban2010 [41] |
| LR | 30 | 2.4 | 30 | 72 | 41 | 0 + | 0.970 [c] | Kuban2010 [41] |
| LR | 157 | 2 | 37 | 74 | 50 | 81.5 | 0.968 [dd] | Dearnaley2016 [34] |
| []LR | 164 | 3 | 20 | 60 | 27 | 83.5 | 0.968 [dd] | Dearnaley2016 [34] |
| LR | 163 | 3 | 19 | 57 | 24 | 79.1 | 0.901 [dd] | Dearnaley2016 [34] |
| LR | 542 | 1.8 | 41 | 73.8 | 56 | 0 | 0.919 [c] | Lee2016 [43] |
| LR | 550 | 2.5 | 28 | 70 | 37 | 0 | 0.937 [c] | Lee2016 [43] |
| LR | 18 | 8 | 5 | 40 | 29 | 0 | 1 [c] | Alayed2018 [29] |
| LR | 136 | 7 | 5 | 35 | 7 | 0 | 0.963 [cc] | Levin-Epstein2021 [44] |

| | | | | | | | | |
|---|---|---|---|---|---|---|---|---|
| LR | 365 | 7.25 | 5 | 36.25 | 7 | 0 | 0.965 [cc] | Levin-Epstein2021 [44] |
| LR | 353 | 8 | 5 | 40 | 7 | 0 | 0.991 [cc] | Levin-Epstein2021 [44] |
| LR | 100 | 9.5 | 4 | 38 | 7 | 0 | 0.991 [cc] | Levin-Epstein2021 [44] |
| | | | | | | | | |
| IR | 215 | 7.25 | 5 | 36.25 | 0 | 10.7 | 0.875 [a] | Davis2015 [33] |
| IR | 12 | 9 | 5 | 45 | 0 | 0 | 0.885 [c] | Hannan2016 [36] |
| IR | 7 | 9.5 | 5 | 47.5 | 0 | 0 | 1 [c] | Hannan2016 [36] |
| IR | 39 | 10 | 5 | 50 | 0 | 0 | 1 [c] | Hannan2016 [36] |
| IR | 12 | 8 | 5 | 40 | 29 | 0 | 0.917 [c] | Alayed2018 [29] |
| IR | 39 | 9.5 | 4 | 38 | 0 | 0 | 0.920 [e] | Fuller2014 [35] |
| IR | 10 | 8.5 | 4 | 34 | 3 | 100 | 1 [c] | Kang2011 [38] |
| IR | 153 | 7.25 | 5 | 36.25 | 4 | 22 * | 0.913 [a] | Katz2016 [39] |
| IR | 50 | 7.25 | 5 | 36.25 | 9.7 | 37.7 | 0.942 [bb] | Bernetich2014 [31] |
| IR | 839 | 3.13 | 16 | 50 | 21 | 38.5 | 0.607 [e] | Miralbell2012 (Logue data) [4] |
| IR | 30 | 4 | 14 | 56 | 28 | 0 | 0.720 [e] | Miralbell2012 [4] |
| IR | 253 | 2 | 39 | 78 | 52 | 55.3 | 0.852 [e] | Kupelian2007/Miralbell2012 [42, 4] |
| IR | 318 | 2.5 | 28 | 70 | 37 | 66.0 | 0.840 [e] | Kupelian2007/Miralbell2012 [42, 4] |
| IR | 278 | 2 | 33 | 66 | 44 | 0 | 0.380 [e] | Lukka2005/Miralbell2012 [45, 4] |
| IR | 265 | 2.63 | 20 | 52.5 | 27 | 0 | 0.470 [e] | Lukka2005/Miralbell2012 [45, 4] |
| IR | 63 | 2 | 32 | 64 | 43 | 0 | 0.570 [e] | Yeoh2006/Miralbell2012 [49, 4] |
| IR | 57 | 2.75 | 20 | 55 | 27 | 0 | 0.670 [e] | Yeoh2006/Miralbell2012 [49, 4] |
| IR | 322 | 1.8 | 45 | 81 | 62 | 86 ** | 0.859 [aa]<br>(55 months) | Zelefsky2002 [50] |
| IR | 324 | 2 | 38 | 76 | 51 | 33.3 | 0.873 [e] | Miralbell2012 (Leborgne data) [4] |
| IR | 71 | 1.8 | 42 | 75.6 | 57 | 28 *** | 0.900 [c] | Kuban2010 [41] |
| IR | 71 | 2.4 | 30 | 72 | 41 | 32 + | 0.950 [c] | Kuban2010 [41] |
| IR | 104 | 3.4 | 19 | 64.6 | 42 | 68 ++ | 0.875 [ee] | Incrocci2016 [37] |
| IR | 107 | 2 | 39 | 78 | 52 | 68 ++ | 0.855 [ee] | Incrocci2016 [37] |
| IR | 779 | 2 | 37 | 74 | 50 | 100 | 0.867 [dd] | Dearnaley2016 [34] |
| IR | 784 | 3 | 20 | 60 | 27 | 100 | 0.898 [dd] | Dearnaley2016 [34] |
| IR | 784 | 3 | 19 | 57 | 24 | 100 | 0.858 [dd] | Dearnaley2016 [34] |
| IR | 598 | 2 | 39 | 78 | 52 | 0 | 0.850 [dd] | Catton2017 [32] |
| IR | 608 | 3 | 20 | 60 | 27 | 0 | 0.850 [dd] | Catton2017 [32] |
| IR | 120 | 7 | 5 | 35 | 7 | 0 | 0.89 [cc] | Levin-Epstein2021 [44] |
| IR | 346 | 7.25 | 5 | 36.25 | 7 | 0 | 0.952 [cc] | Levin-Epstein2021 [44] |
| IR | 331 | 8 | 5 | 40 | 7 | 0 | 0.92 [cc] | Levin-Epstein2021 [44] |
| IR | 157 | 9.5 | 4 | 38 | 7 | 0 | 0.836 [cc] | Levin-Epstein2021 [44] |
| | | | | | | | | |
| HR | 29 | 8.5 | 4 | 34 | 3 | 100 | 0.908 [c] | Kang2011 [38] |

| | | | | | | | | |
|---|---|---|---|---|---|---|---|---|
| HR | 38 | 7.25 | 5 | 36.25 | 4 | 100 * | 0.791 [a] | Katz2016 [39] |
| HR | 31 | 7.25 | 5 | 36.25 | 9.7 | 50 | 0.839 [bb] | Bernetich2014 [31] |
| HR | 821 | 3.13 | 16 | 50 | 21 | 50.2 | 0.560 [e] | Miralbell2012 (Logue data) [4] |
| HR | 20 | 4 | 14 | 56 | 28 | 0 | 0.740 [e] | Miralbell2012 [4] |
| HR | 83 | 3.1 | 20 | 62 | 34 | 100 | 0.858 [bb] | Arcangeli2012/2017 [14, 30] |
| HR | 85 | 2 | 40 | 80 | 55 | 100 | 0.788 [bb] | Arcangeli2012/2017 [14, 30] |
| HR | 233 | 2 | 39 | 78 | 52 | 97.4 | 0.747 [e] | Kupelian2007/Miralbell2012 [42, 4] |
| HR | 217 | 2.5 | 28 | 70 | 37 | 98.2 | 0.650 [e] | Kupelian2007/Miralbell2012 [42, 4] |
| HR | 79 | 2 | 33 | 66 | 44 | 0 | 0.280 [e] | Lukka2005/Miralbell2012 [45, 4] |
| HR | 88 | 2.63 | 20 | 52.5 | 27 | 0 | 0.290 [e] | Lukka2005/Miralbell2012 [45, 4] |
| HR | 12 | 2 | 32 | 64 | 43 | 0 | 0.420 [e] | Yeoh2006/Miralbell2012 [49, 4] |
| HR | 25 | 2.75 | 20 | 55 | 27 | 0 | 0.640 [e] | Yeoh2006/Miralbell2012 [49, 4] |
| HR | 175 | 1.8 | 45 | 81 | 62 | 100 ** | 0.808 [aa] (56 months) | Zelefsky2002 [50] |
| HR | 295 | 2 | 38 | 76 | 51 | 55.9 | 0.664 [e] | Miralbell2012 (Leborgne data) [4] |
| HR | 303 | 3.4 | 19 | 64.6 | 42 | 68 ++ | 0.779 [ee] | Incrocci2016 [37] |
| HR | 290 | 2 | 39 | 78 | 52 | 68 ++ | 0.740 [ee] | Incrocci2016 [37] |
| HR | 129 | 2 | 37 | 74 | 50 | 100 | 0.866 [dd] | Dearnaley2016 [34] |
| HR | 126 | 3 | 20 | 60 | 27 | 100 | 0.841 [dd] | Dearnaley2016 [34] |
| HR | 130 | 3 | 19 | 57 | 24 | 100 | 0.788 [dd] | Dearnaley2016 [34] |

**Definition of control**

[a] biochemical disease free survival

[b] biochemical progression free survival

[c] biochemical failure free survival

[d] biochemical freedom from relapse

[e] biochemical relapse free survival

[aa] PSA relapse free survival

[bb] freedom from biochemical failure

[cc] biochemical recurrence free survival

[dd] biochemical or clinical free survival (data were included because biochemical failure represented most of the failure events, ~90%)

[ee] relapse free survival: biochemical, clinical, locoregional or distant failure (data were included because biochemical failure represented most of the failure events, ~90%)

**ADT status**

* 72 patients (14%) out of 515 (324 LR, 153 IR, 38 HR) received ADT. Because ADT is most likely prescribed to HR/IR patients, we assume that 38/38 (100%) HR, 34/153 (22%) IR and 0/324 (0%) LR patients received ADT.

** 55% of 772 patients (275 LR, 322 IR, 175 HR) received ADT. Because ADT is most likely prescribed to HR/IR patients, we assume that 175/175 (100%) HR, 250/ 322(78%) IR and 0/275 (0%) LR patients received ADT.

*** 20 patients out of 101 (30 LR, 71 IR) received ADT. We assume that all of them belong to the IR group and none to the LR group.

+ 23 patients out of 101 (30 LR, 71 IR) received ADT. We assume that all of them belong to the IR group and none to the LR group.

++ 537/804 patients in this study involving IR and HR patients received ADT. Separated percentages for IR and HR are not provided, and we have considered 68% for each group.
